# Identification of *Penicillium* species by MALDI-TOF MS analysis of spores collected by dielectrophoresis

**DOI:** 10.1093/biomethods/bpz018

**Published:** 2019-11-29

**Authors:** Michael A Reeve, Denise Bachmann, Thelma S Caine

**Affiliations:** CABI, Egham, UK

**Keywords:** fungal-spore enrichment, dielectrophoresis, triboelectric charging, protein extraction, fungal identification

## Abstract

In matrix-assisted laser-desorption and ionization mass spectrometry, spectral differences are frequently observed using different growth media on agar plates and/or different growth times in culture, which add undesirable analytical variance. In this article, we explore an approach to the above problem based upon the rationale that, while protein expression in fungal mycelium may well vary under different growth conditions, this might not apply to the same extent in fungal spores. To this end, we have exploited the fact that while mycelium is generally anchored to the fungal-growth substrate, some fungi produce physically-isolated spores which, as such, are amenable to manipulation using dielectrophoresis (the translational motion of charged or uncharged matter caused by polarization effects in a non-uniform electrical field). Such fields can be conveniently generated through the charging of an insulator using the triboelectric effect (the transfer of charge between two objects through friction when they are rubbed together). In this study, polystyrene microbiological inoculating loops were used in combination with nylon-fabric rubbing to harvest fungal spores from five species from within the genus *Penicillium*, which were grown on agar plates containing two different media over an extended time course. In terms of average Bruker spectral-comparison scores, our method generated higher scores in 80% of cases tested and, in terms of average coefficients of variation, our method generated lower spectral variability in 93% of cases tested. Harvesting of spores using a rapid, inexpensive and simple dielectrophoretic method, therefore, facilitates improved fungal identification for the *Penicillium* species tested.

## Introduction

Characterization and/or identification of biological samples can be achieved through analysis of the mass spectra of their constituent proteins [1, 2]. To this end, one particularly rapid and inexpensive technique is matrix-assisted laser-desorption and ionization mass spectrometry (MALDI-TOF MS) [1, 3]. In MALDI-TOF MS protein ‘fingerprinting’, mass spectra are generally obtained from a subset of the expressed proteome. This is frequently the highly-expressed acid-soluble cellular proteins, a fraction that also includes many ribosomal proteins [2]. For the generation of a MALDI-TOF mass spectrum, proteins are first prepared intact in the gas phase, carrying predominantly single positive charges [4]. Protein desorption and ionization as above are made possible through the laser-initiated ‘MALDI’ soft-ionization process [3]. In the gas phase, such singly-charged proteins may be accelerated using an electrical field [1]. After an initial acceleration in this manner, their times-of-flight along an evacuated tube are proportional to the square root of their mass-over-charge (m/z) ratios [1]. Using this simple mathematical relationship, a mass spectrum can readily be generated for the protein components in a particular biological sample.

Often with a focus on human clinical microbiology, numerous methods have been developed for MALDI-TOF MS sample preparation [1, 5–11]. In an attempt to broaden the analytical scope for MALDI-TOF MS, Reeve *et al*. [12] have also developed a simple and inexpensive method that can additionally be applied to insect [12, 13], plant, [12, 14, 15] and seed material [16, 17]. This latter method lyses cells by immersion (or maceration if required) in aqueous acetonitrile containing trifluoroacetic acid (TFA) to selectively extract acid-soluble proteins, and lysis and extraction are carried out in the presence of near-saturated and inexpensive-grade MALDI matrix. The resulting matrix-saturated lysate (containing acid-solubilized proteins for MALDI-TOF MS analysis) is then simply dried down directly onto the MALDI-TOF MS sample plate. In this article, we have used this method for MALDI-TOF MS sample preparation because of its simplicity and broad applicability.

For the characterization and/or identification of filamentous fungi, significant variations in MALDI-TOF MS spectra are unfortunately observed under different growth conditions on agar plates [18]. As discussed by Reeve and Bachmann [18], the growth medium employed can have a significant influence on protein expression, as can growth time in culture. While such differentiation of fungi on agar plates, along with their adaptation to different growth media, has in the past been exploited to aid their characterization and/or identification [19], these add undesirable variance to MALDI-TOF MS-based analyses. In order to minimize such variance, we have previously investigated fungal growth in small volume liquid culture, along with filter-paper-supported growth in a MALDI-TOF MS-compatible rich medium within sealed 1.5 ml Eppendorf tubes, carrying out protein extraction from the entire culture in each case. Using the latter method, we observed slightly better identifications and lower spectral variance compared to agar plate controls [18]. In this article, we explore a complementary approach to the above based upon the speculative rationale that, while protein expression in fungal mycelium may well vary between different growth media and over time in culture, fungal spores might be more invariant to changes in growth media and growth times.

Fungal spores have previously been analysed using MALDI-TOF MS [20]. In these studies, methods for spore preparation have included sucrose density-gradient centrifugation [21, 22], filtration through folded cloth [23], scratching spores directly from the surface of contaminated fruit [24] and washing agar plates with 0.1% (v/v) Tween-20 [25]. For the routine and/or high-throughput identification of suitable fungi via their spores, there would be a considerable benefit if a more convenient, rapid and the inexpensive method was available for the collection of relatively-pure spores for analysis using MALDI-TOF MS. In order to develop such a method, a ‘target property’ of fungal spores that distinguishes them from vegetative mycelium might be considered as a useful starting point. To this end, fungal spores cover a variety of types, shapes and sizes (which may conveniently be exploited for fungal classification and/or identification [19]) and, as they vary from species to species, fungal spores are unlikely to have common features of surface chemistry that can be exploited for their separation from mycelium. Not all species of fungi form well-developed aerial mycelium during cultivation but, even if mycelium is deeply anchored to the growth substrate, their asexual spores, when mature, are nevertheless physically-isolated entities – microscopic particles separated from the mycelium. As such, they should, therefore, be amenable to manipulation using dielectrophoresis (DEP) [26], a phenomenon for which the theoretical basis is comprehensively reviewed by Pethig [27], along with numerous applications of DEP within microfluidic devices.

As background, two processes may operate when a particle is placed in an electrical field. These are electrophoresis (the translational motion of charged matter in an electrical field, which may be either uniform or non-uniform) and DEP (the translational motion of charged or uncharged matter caused by polarization effects in a non-uniform electrical field). Electrical fields can interact with matter in two ways, namely through conduction (where charges move freely through the particle) and through polarization (where charge movement is constrained). In a non-uniform electrical field, two types of force may be exerted on a particle within the field. Particles with an overall net charge will be pulled along the field lines towards the electrode with the opposite sign to the charge on the particle. The non-uniform electrical field will, however, also induce polarization in the particle and, because such a field has a different strength on one side of the particle compared to the other, a net force will be exerted. This net force will move the particle towards regions of higher field strength (the ‘paraelectric’ effect). Fairly divergent electrical fields are generally required for DEP, along with high field strengths and larger particles experience a greater net force in a non-uniform electrical field (because the field-strength difference across the particle will be greater). The net force is also proportional to the particle polarizability and to the square of the electrical field. DEP requires that there is a difference in dielectric constant between the medium and the particle, and the net force is greater when there is a greater difference between these [27, 28].

A convenient way of generating a non-uniform electrical field is through the charging of an insulating rod (e.g. a plastic microbiological inoculating loop) using the triboelectric effect, which is the transfer of charge between two objects through friction when they are rubbed together. The amount of charge transferred depends on the electron affinities of the materials [29], with the material having the higher electron affinity becoming negatively charged. In this study, polystyrene plating loops were used in combination with nylon-fabric rubbing.

In this article, we investigate the potential for identification of fungi by MALDI-TOF MS analysis of spores collected by DEP and using the above highly-simplified and inexpensive method for MALDI-TOF MS sample preparation [12], employing representative strains of fungi from the genus *Penicillium* that were readily available to us from the CAB International (CABI) Genetic Resource Collection [30, 31], with growth on agar plates containing two different media over an extended time course.

## Materials and methods

### Fungal strains 

*Penicillium chrysogenum* [Thom], IMI 293188; *P.* *corylophilum* [Dierckx], IMI 273248; *P.* *digitatum* [(Pers.) Sacc.], IMI 380881; *P.* *glabrum* [(Wehmer) Westling], IMI 320720; and *P.* *roqueforti* [Thom], IMI 297987 were chosen for investigation in this study.

### Fungal growth

Potato dextrose agar (PDA) was made up of 4 g/l potato extract, 20 g/l glucose and 15 g/l agar, adjusted to pH 5.6. Malt-extract agar (MEA) was made up of 20 g/l malt extract, 1 g/l bacteriological peptone, 20 g/l glucose and 15 g/l agar. Cultures were grown for 3, 6, 10 and 17 days on 5 cm plates at 25°C.

### Reagents and inoculating loops

Greater than or equal to 98% (TLC-grade) *α*-cyano-4-hydroxycinnamic acid (HCCA) matrix, LC-MS-grade acetonitrile and 99% ReagentPlus^®^-grade TFA, were purchased from Sigma (Gillingham, UK). CHROMASOLV^™^ LC-MS-grade water was purchased from Fluka (Loughborough, UK). One microliter dark-green COPAN inoculating loops were purchased from Thermo Fisher Scientific (Loughborough, UK).

### MALDI-TOF MS sample preparation

For acid-soluble-protein preparations from mixed spore-and-mycelium fungal cultures grown on agar plates (abbreviated CON below), biomass was carefully scraped from the agar and mixed with 60 µl of Extraction Reagent [11 mg/ml HCCA matrix in 65% (v/v) acetonitrile, 2.5% (v/v) TFA and 32.5% (v/v) water] using a plastic inoculating loop. One microliter of the resulting crude lysate was then pipetted onto the Bruker sample plate, air dried and loaded into the spectrometer. For acid-soluble-protein preparations from spores harvested using DEP (abbreviated DEP below), a plastic inoculating loop was briefly rubbed around the terminal loop with a nylon cloth and the loop was then placed in gentle contact with the sporulating-biomass surface using each side of the loop to collect spores by DEP. The spore-coated plastic was then immersed in 60 µl of Extraction Reagent, with the rotation of the loop-end for around 20 s. One microliter of the resulting crude lysate was again pipetted onto the Bruker sample plate, air-dried and loaded into the spectrometer.

### Mass spectrometry

Mass spectrometry covering the range 2–20 kDa was carried out using a Bruker Microflex LT linear-mode instrument running the MALDI Biotyper 4.0 applications (Bruker Daltonik, Bremen, Germany), using a 60 Hz frequency and 3 ns pulse-duration nitrogen laser (70 µJ, with maximum output 225 µJ), with a wavelength of 337 nm and spot size of 100 µm, with 240 laser shots per sample. The laser settings were global attenuator offset (0%), attenuator offset (22%) and attenuator range (30%), and the ion-source voltage was 19.98 kV. Bruker MBT Biotarget 96 plates (Bruker ref. 1840375) were used for all samples in this study. Calibration was carried out using the manufacturer’s ‘Bacterial Test Standard’ controls (*Escherichia coli* proteins supplemented with ribonuclease A and myoglobin), using peaks with masses at 3637.8; 5096.8; 5381.4; 6255.4; 7274.5; 10 300.2; 13 683.2; and 16 952.3 for calibration according to the manufacturer’s instructions. Spectra were acquired using MALDI Biotyper RTC version 4.0 (Build 19) using the manufacturer’s standard settings (Centroid peak-detection algorithm and TopHat baseline subtraction). Database entries were made as single-spectra Main Spectra (MSPs) using the Bruker Online Client software suite version 4.0.19 (Bruker Daltonik, Bremen, Germany), again using the manufacturer’s standard settings. For spectral comparisons, Bruker identification scores were derived using the standard Bruker algorithm. This first converts raw mass spectra into peak lists, which are then compared between spectra. Three separate values are computed: the number of peaks in the reference spectrum that have a closely-matching partner in the test spectrum (value range 0–1), the number of peaks in the test spectrum that have a closely-matching partner in the reference spectrum (value range 0–1) and the peak-height symmetry of the matching peaks (value range 0–1). The above three values are multiplied together and normalized to 1000, and the base-10 logarithm is then taken to give the final Bruker score (range 0–3). Bruker scores between 2.3 and 3.0 indicate very close relatedness, scores between 2.0 and 2.3 indicate close relatedness, scores between 1.7 and 2.0 indicate intermediate relatedness and scores <1.7 indicate low relatedness. All spectra are shown baseline subtracted, smoothed, *y*-axis-autoscaled and covering the mass range 2–20 kDa (with *x*-axis scale increments of 2 kDa).

### Spectral comparisons

Triplicate sample preparations, from single agar plates, were carried out for each strain and culture-condition combination at 3, 6, 10 and 17 days incubation at 25°C. Replicate-1 sample preparations for each strain and culture-condition combination were used to construct a custom database of ‘reference’ mass spectra (as single-spectra MSPs using the Bruker Online Client software) specifically for use in this study. The Replicate-2 and Replicate-3 ‘test’ samples were then compared to these Replicate-1 reference spectra from the custom database, again using the Bruker Online Client software suite as described above. We have again used the ‘single-spectrum MSP’ approach discussed in Reeve and Seehausen [13], which departs slightly from the ‘standard Bruker method’ for routine clinical identifications of bacteria and yeasts in that every single spectrum obtained is used to make a separate database entry, and all variance in spectral comparisons can thereby be displayed in the results, rather than being subsumed into an averaged spectral construct.

## Results

**Figure 1:**
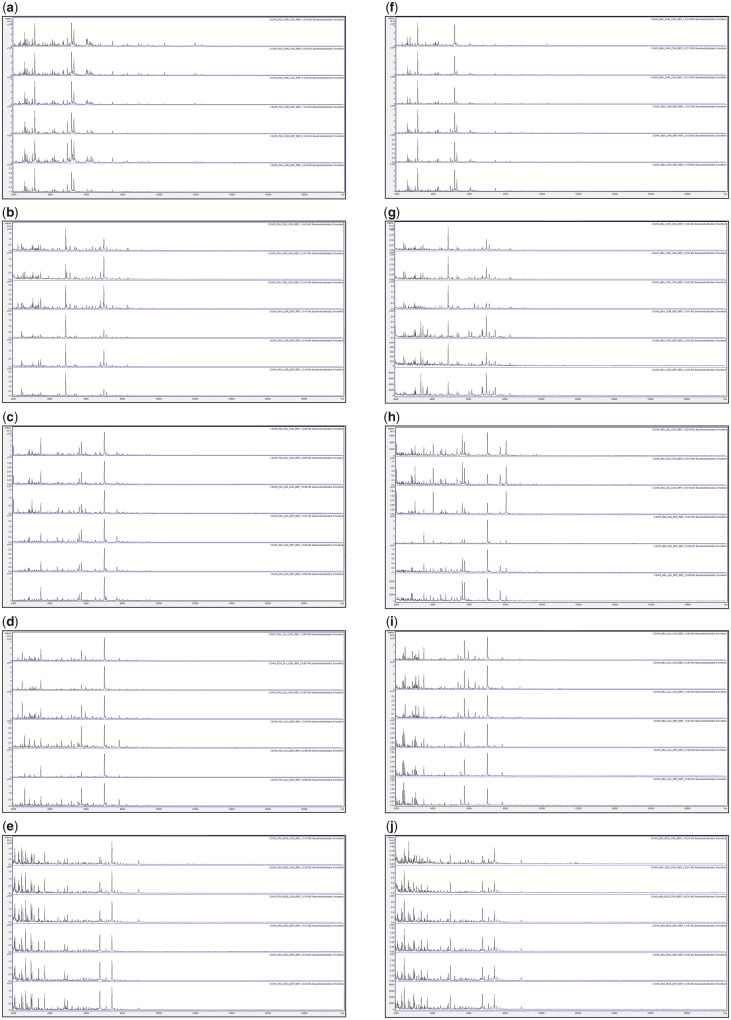
MALDI-TOF MS spectra obtained after growth for 3 days at 25°C with, from top to bottom in each panel, all biomass scraped from the agar (Replicates 1, 2 and 3) and spores collected by DEP (Replicates 1, 2 and 3). The species panels are (**a**, **f**) *P. chrysogenum* IMI 293188, (**b**, **g**) *P. corylophilum* IMI 273248, (**c**, **h**) *P. digitatum* IMI 380881, (**d**, **i**) *P. glabrum* IMI 320720 and (**e**, **j**) *P. roqueforti* IMI 297987. The left-hand panels (a–e) are for growth on PDA plates and the right-hand panels (f–j) are for growth on MEA plates.

**Figure 2:**
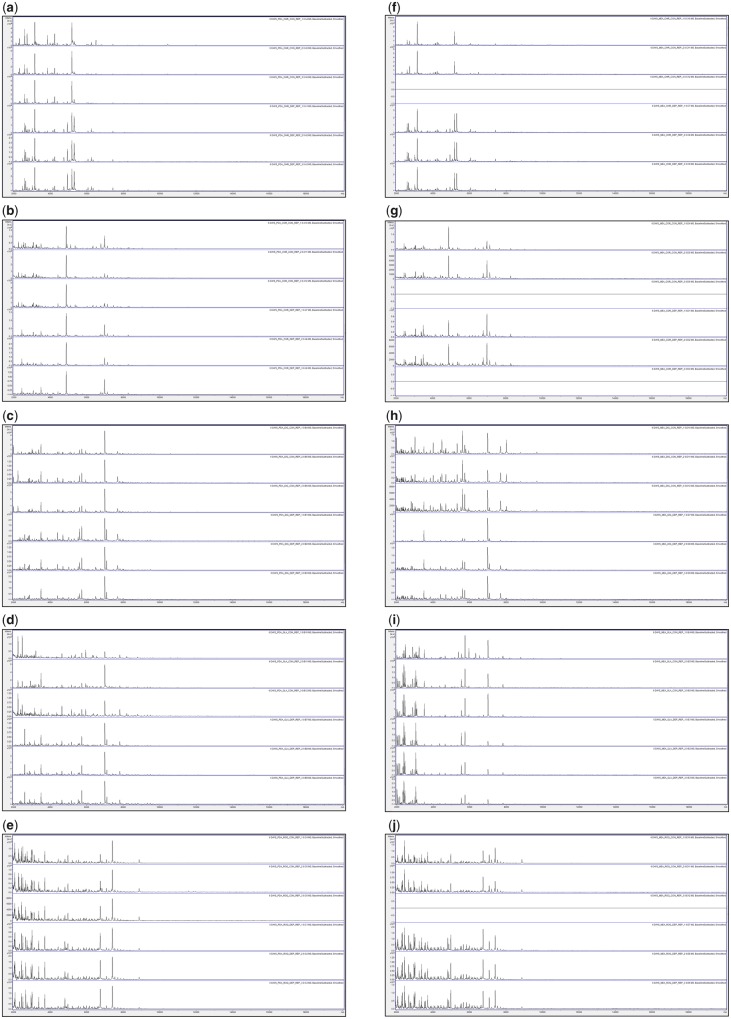
MALDI-TOF MS spectra obtained after growth for 6 days at 25°C with, from top to bottom in each panel, all biomass scraped from the agar (Replicates 1, 2 and 3) and spores collected by DEP (Replicates 1, 2 and 3). The species panels are (**a**, **f**) *P. chrysogenum* IMI 293188, (**b**, **g**) *P. corylophilum* IMI 273248, (**c**, **h**) *P. digitatum* IMI 380881, (**d**, **i**) *P. glabrum* IMI 320720 and (**e**, **j**) *P. roqueforti* IMI 297987. The left-hand panels (a–e) are for growth on PDA plates and the right-hand panels (f–j) are for growth on MEA plates.

**Figure 3:**
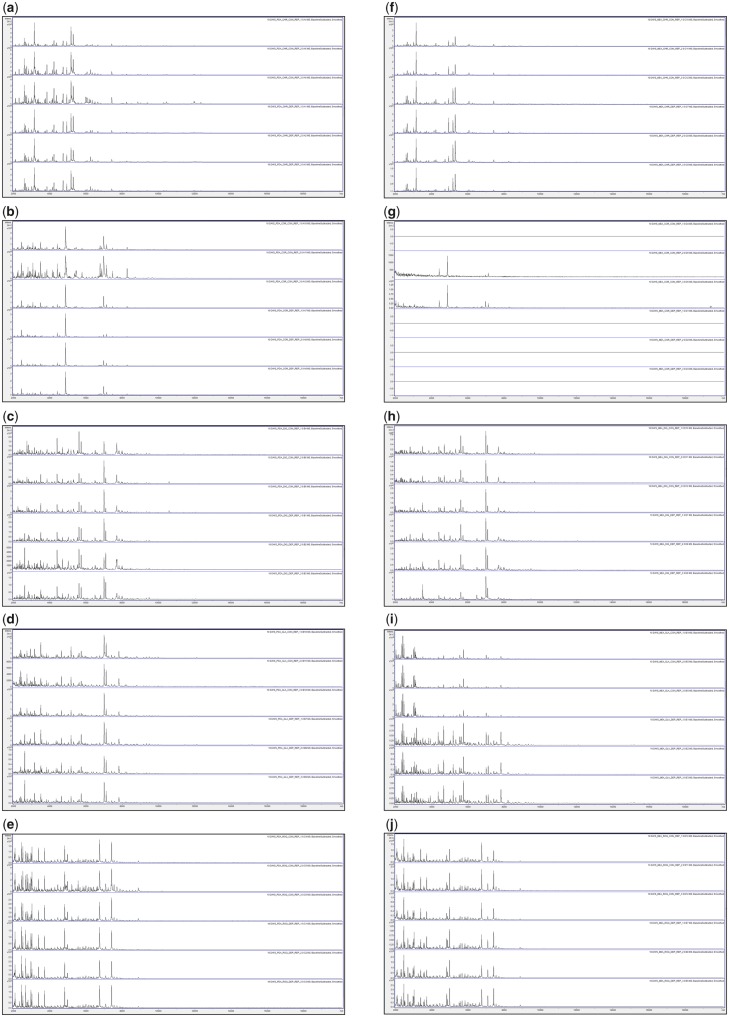
MALDI-TOF MS spectra obtained after growth for 10 days at 25°C with, from top to bottom in each panel, all biomass scraped from the agar (Replicates 1, 2 and 3) and spores collected by DEP (Replicates 1, 2 and 3). The species panels are (**a**, **f**) *P. chrysogenum* IMI 293188, (**b**, **g**) *P. corylophilum* IMI 273248, (**c**, **h**) *P. digitatum* IMI 380881, (**d**, **i**) *P. glabrum* IMI 320720 and (**e**, **j**) *P. roqueforti* IMI 297987. The left-hand panels (a–e) are for growth on PDA plates and the right-hand panels (f–j) are for growth on MEA plates.

**Figure 4:**
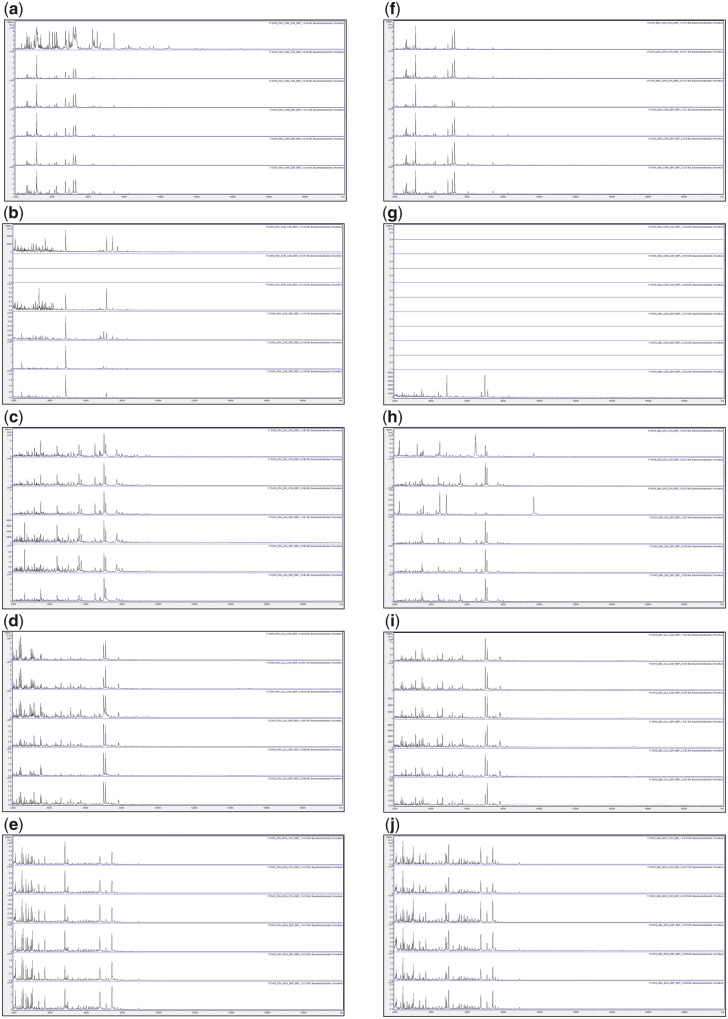
MALDI-TOF MS spectra obtained after growth for 17 days at 25°C with, from top to bottom in each panel, all biomass scraped from the agar (Replicates 1, 2 and 3) and spores collected by DEP (Replicates 1, 2 and 3). The species panels are (**a**, **f**) *P. chrysogenum* IMI 293188, (**b**, **g**) *P. corylophilum* IMI 273248, (**c**, **h**) *P. digitatum* IMI 380881, (**d**, **i**) *P. glabrum* IMI 320720 and (**e**, **j**) *P. roqueforti* IMI 297987. The left-hand panels (a–e) are for growth on PDA plates and the right-hand panels (f–j) are for growth on MEA plates.

Figures 1–4 show the triplicate MALDI-TOF MS spectra obtained after growth for 3, 6, 10 and 17 days at 25°C, respectively, with all biomass scraped from the agar, and spores collected by DEP, for *P.* *chrysogenum* IMI 293188, *P.* *corylophilum* IMI 273248, *P.* *digitatum* IMI 380881, *P.* *glabrum* IMI 320720 and *P.* *roqueforti* IMI 297987, with growth on PDA plates and growth on MEA plates. Triplicate negative controls for the DEP collection method (but with no contact with spores) were also carried out and gave no spectra (Supplementary Fig. S1).

Figure 1 shows very good spectral replication after 3 days of growth between triplicates for all samples except *P. chrysogenum* IMI 293188 grown on PDA, *P. glabrum* IMI 320720 grown on PDA and *P. digitatum* IMI 380881 grown on MEA, where some variation in peak height is observed for the spectra obtained using both methods. In most cases, the spectra obtained by both methods are visibly similar, though noticeably more peaks are obtained from *P. corylophilum* IMI 273248 grown on MEA for biomass scraped from the agar.

Figure 2 shows very good spectral replication after 6 days of growth between triplicates for all samples except *P. glabrum* IMI 320720 grown on PDA for biomass scraped from the agar, where some variation in peak height is observed. In most cases, the spectra obtained by both methods are visibly similar, though noticeably more peaks are obtained from *P. digitatum* IMI 380881 grown on MEA for biomass scraped from the agar. No spectra were obtained for *P. chrysogenum* IMI 293188 Replicate-3 grown on MEA for biomass scraped from the agar, *P. corylophilum* IMI 273248 Replicate-3 grown on MEA for biomass scraped from the agar, *P. corylophilum* IMI 273248 Replicate-3 grown on MEA for spores collected by DEP and *P. roqueforti* IMI 297987 Replicate-3 grown on MEA for biomass scraped from the agar.

Figure 3 shows very good spectral replication after 10 days of growth between triplicates for all samples except *P. chrysogenum* IMI 293188 grown on PDA for biomass scraped from the agar and *P. corylophilum* IMI 273248 grown on PDA for biomass scraped from the agar, where some variation in peak height is observed. In most cases, the spectra obtained by both methods are visibly similar, though noticeably more peaks are obtained from *P. glabrum* IMI 320720 grown on MEA for spores collected by DEP. No spectra were obtained for *P. corylophilum* IMI 273248 Replicate-1 grown on MEA for biomass scraped from the agar and *P. corylophilum* IMI 273248 Replicates 1, 2 and 3 grown on MEA for spores collected by DEP.

Figure 4 shows very good spectral replication after 17 days of growth between triplicates for all samples except *P. chrysogenum* IMI 293188 grown on PDA for biomass scraped from the agar, *P. corylophilum* IMI 273248 grown on PDA for biomass scraped from the agar, *P. corylophilum* IMI 273248 grown on PDA for spores collected by DEP, and *P. digitatum* IMI 380881 grown on MEA for biomass scraped from the agar, where some variation in peak height is observed (particularly in the latter case). In most cases, the spectra obtained by both methods are visibly similar with the exception of the abovementioned samples. No spectra were obtained for *P. corylophilum* IMI 273248 Replicate-2 grown on PDA for biomass scraped from the agar, *P. corylophilum* IMI 273248 Replicates 1, 2 and 3 grown on MEA for biomass scraped from the agar, and *P. corylophilum* IMI 273248 Replicates 1 and 2 grown on MEA for spores collected by DEP.

**Figure 5:**
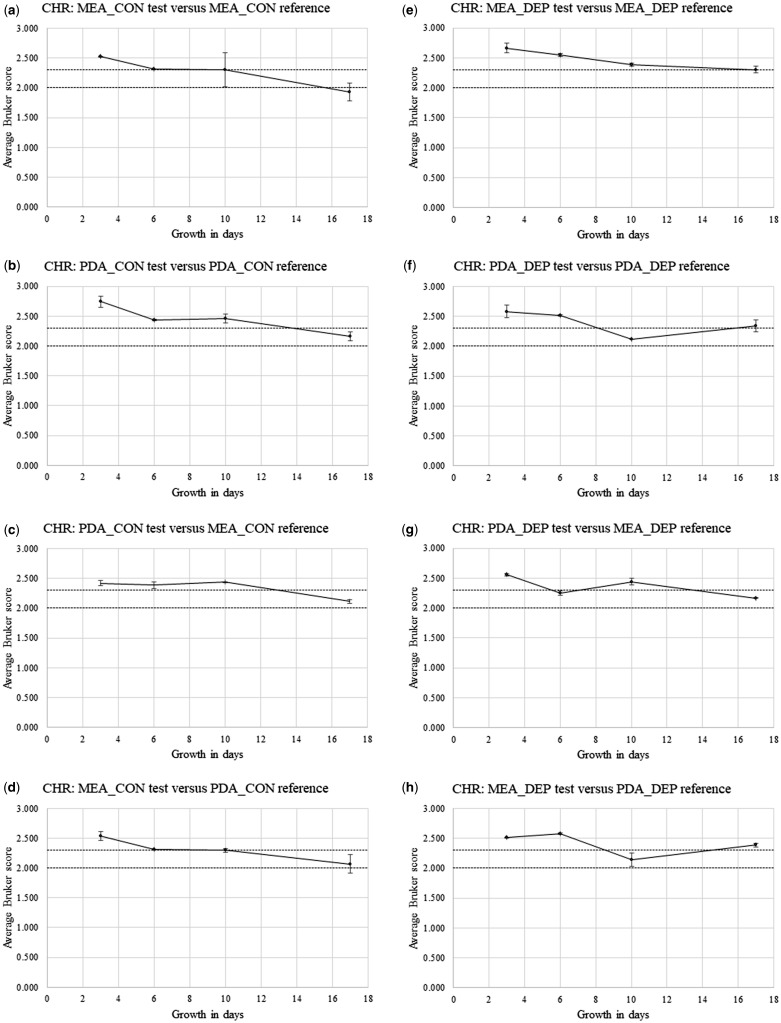
Average Bruker scores (with error bars indicating 1 SD either side of the mean) for comparison between Replicate-2 and Replicate-3 test sample spectra at 3, 6, 10 and 17 days and Replicate-1 reference sample spectra at 3 days for *P. chrysogenum* IMI 293188, with (**a**, **b**, **e** and **f**) cognate-media comparisons and (**c**, **d**, **g** and **h**) non-cognate-media comparisons and with (a–d) biomass scraped from the agar (CON) and (e–h) spores collected by DEP (DEP). The Bruker-score boundaries for close relatedness (2.0) and very close relatedness (2.3) are indicated by broken horizontal lines.

**Figure 6:**
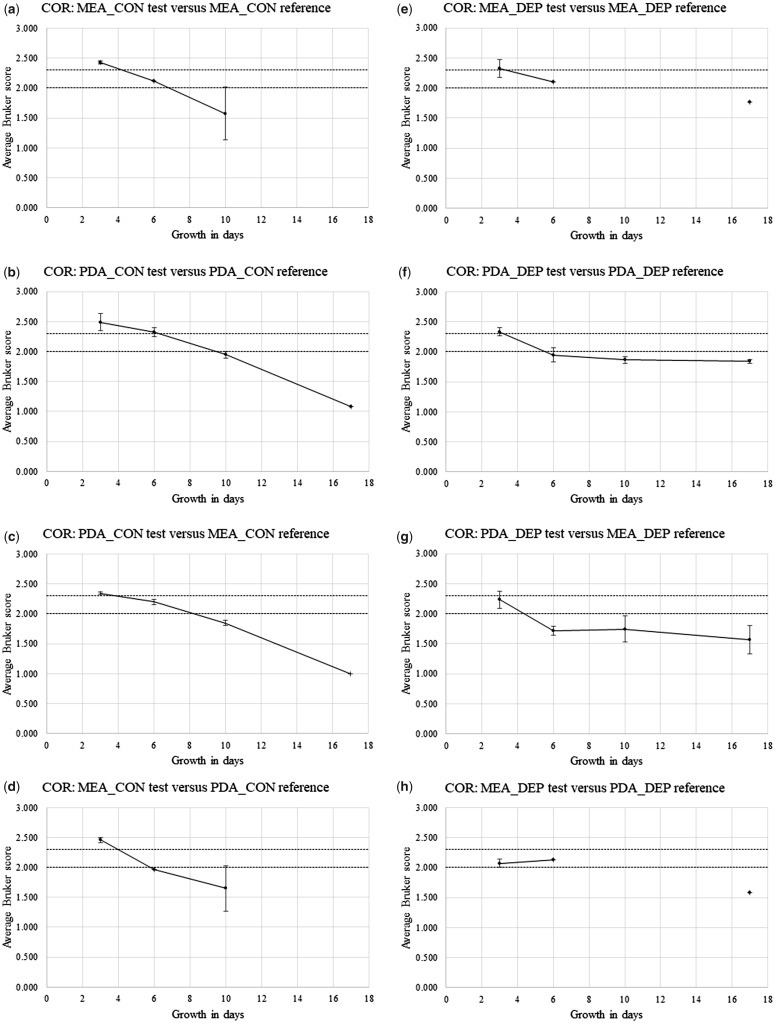
Average Bruker scores (with error bars indicating 1 SD either side of the mean) for comparison between Replicate-2 and Replicate-3 test sample spectra at 3, 6, 10 and 17 days and Replicate-1 reference sample spectra at 3 days for *P. corylophilum* IMI 273248, with (**a**, **b**, **e** and **f**) cognate-media comparisons and (**c**, **d**, **g** and **h**) non-cognate-media comparisons and with (a–d) biomass scraped from the agar (CON) and (e–h) spores collected by DEP (DEP). The Bruker score boundaries for close relatedness (2.0) and very close relatedness (2.3) are indicated by broken horizontal lines.

**Figure 7:**
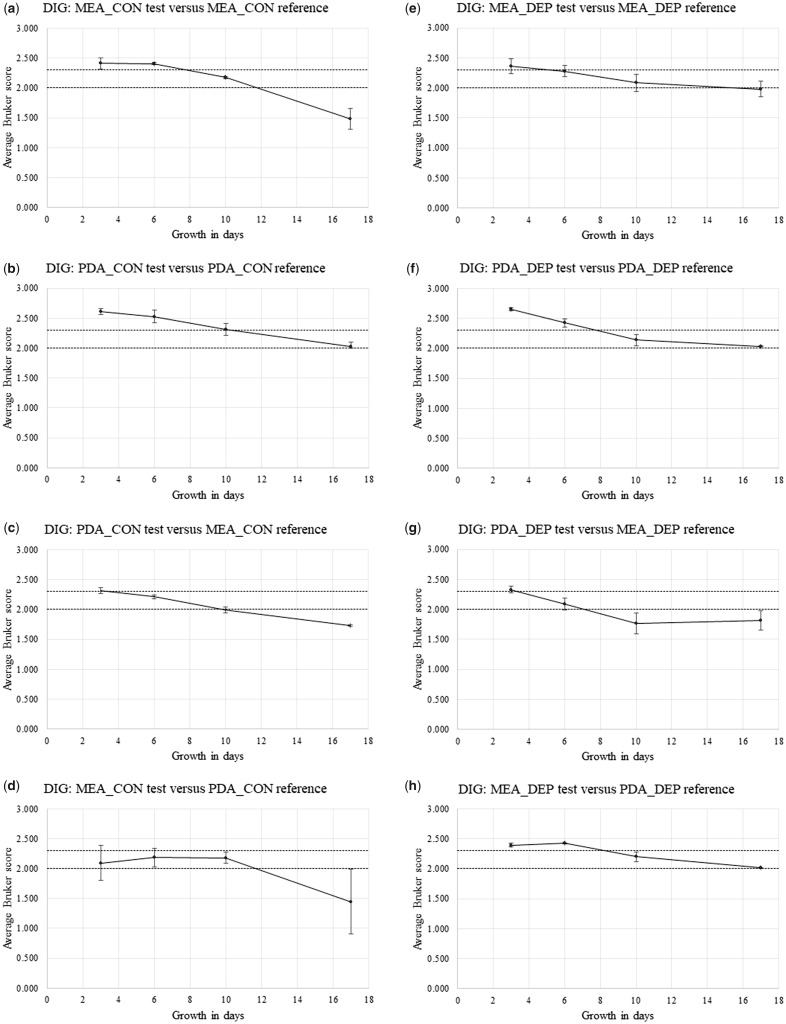
Average Bruker scores (with error bars indicating 1 SD either side of the mean) for comparison between Replicate-2 and Replicate-3 test sample spectra at 3, 6, 10 and 17 days and Replicate-1 reference sample spectra at 3 days for *P. digitatum* IMI 380881, with (**a**, **b**, **e** and **f**) cognate-media comparisons and (**c**, **d**, **g** and **h**) non-cognate-media comparisons and with (a–d) biomass scraped from the agar (CON) and (e–h) spores collected by DEP (DEP). The Bruker-score boundaries for close relatedness (2.0) and very close relatedness (2.3) are indicated by broken horizontal lines.

**Figure 8:**
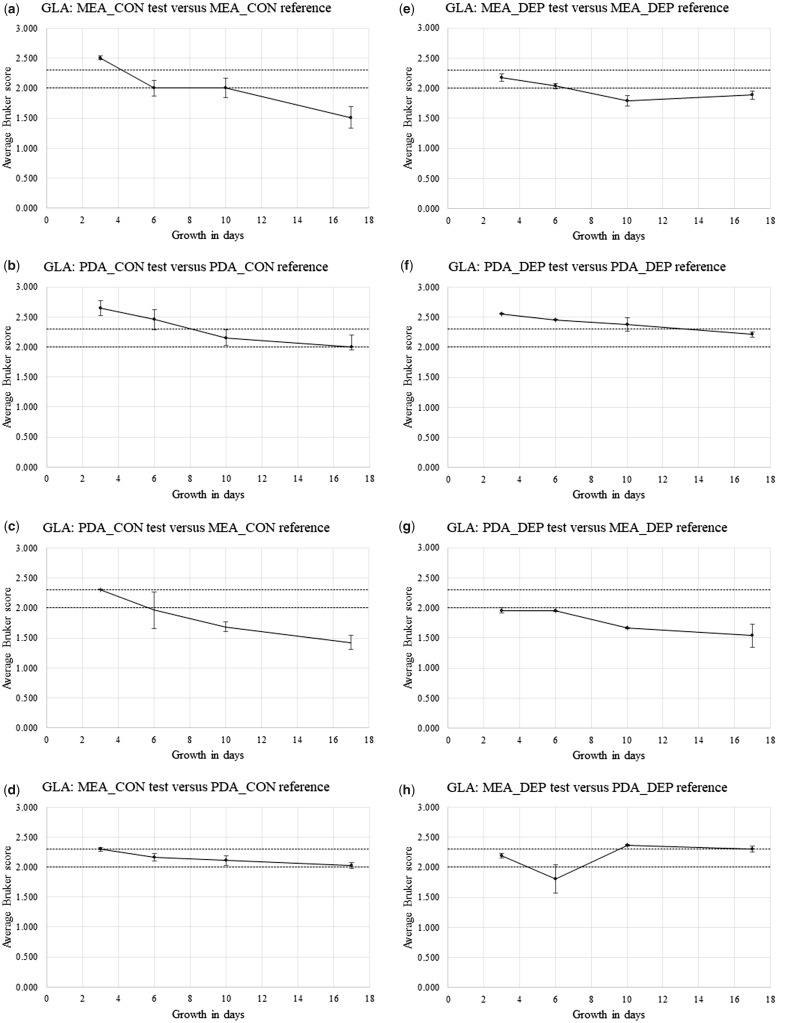
Average Bruker scores (with error bars indicating 1 SD either side of the mean) for comparison between Replicate-2 and Replicate-3 test sample spectra at 3, 6, 10 and 17 days and Replicate-1 reference sample spectra at 3 days for *P. glabrum* IMI 320720, with (**a**, **b**, **e** and **f**) cognate-media comparisons and (**c**, **d**, **g** and **h**) non-cognate-media comparisons and with (a–d) biomass scraped from the agar (CON) and (e–h) spores collected by DEP (DEP). The Bruker score boundaries for close relatedness (2.0) and very close relatedness (2.3) are indicated by broken horizontal lines.

**Figure 9:**
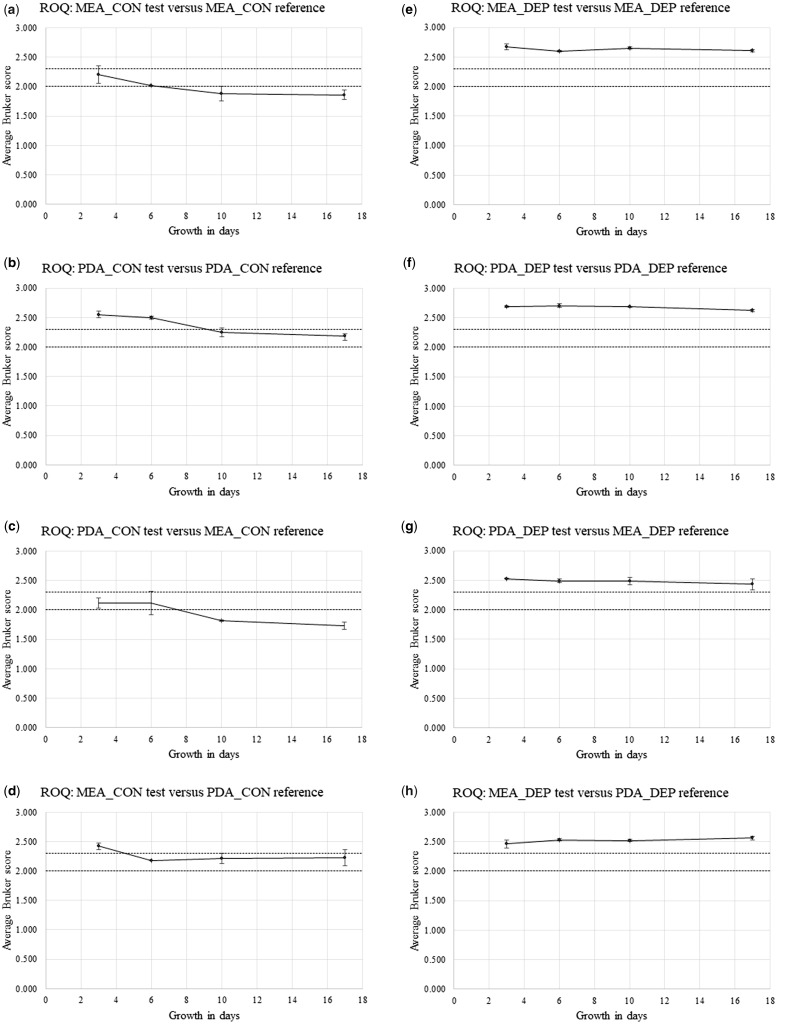
Average Bruker scores (with error bars indicating 1 SD either side of the mean) for comparison between Replicate-2 and Replicate-3 test sample spectra at 3, 6, 10 and 17 days and Replicate-1 reference sample spectra at 3 days for *P. roqueforti* IMI 297987, with (**a**, **b**, **e** and **f**) cognate-media comparisons and (**c**, **d**, **g** and **h**) non-cognate-media comparisons and with (a–d) biomass scraped from the agar (CON) and (e–h) spores collected by DEP (DEP). The Bruker score boundaries for close relatedness (2.0) and very close relatedness (2.3) are indicated by broken horizontal lines.

Supplementary Tables S1–S5 show Bruker spectral-comparison scores between Replicate-1 reference-sample spectra and Replicate-2 and Replicate-3 test sample spectra (in alphabetical order) for *P. chrysogenum* IMI 293188 (CHR), *P. corylophilum* IMI 273248 (COR), *P. digitatum* IMI 380881 (DIG), *P. glabrum* IMI 320720 (GLA), and *P. roqueforti* IMI 297987 (ROQ), respectively. Derived from Supplementary Tables S1–S5, Figs. 5–9 show the average Bruker scores (with error bars indicating 1 SD either side of the mean) for comparison between Replicate-2 and Replicate-3 test sample spectra at 3, 6, 10 and 17 days and Replicate-1 reference sample spectra at 3 days for (again in alphabetical order) *P. chrysogenum* IMI 293188, *P. corylophilum* IMI 273248, *P. digitatum* IMI 380881, *P. glabrum* IMI 320720 and *P. roqueforti* IMI 297987, with both cognate-media comparisons and non-cognate-media comparisons, and with both biomass scraped from the agar (CON) and spores collected by DEP (DEP).

Figure 5 shows a small general reduction in spectral similarity over the time course, with noticeably higher average Bruker scores for the MEA cognate-media comparison and spores collected by DEP (Fig. 5e). Figure 6 shows a pronounced general reduction in spectral similarity over the time course for biomass scraped from the agar, with a lesser reduction for spores collected by DEP. Figure 7 shows a general reduction in spectral similarity over the time course for biomass scraped from the agar, with a lesser reduction for spores collected by DEP. Figure 8 shows a small general reduction in spectral similarity over the time course for all cases except the MEA cognate-media comparison and spores collected by DEP (Fig. 8e) and the MEA-test and PDA-reference non-cognate-media comparison and spores collected by DEP (Fig. 8h). Figure 9 shows a small general reduction in spectral similarity over the time course for biomass scraped from the agar but little or no reduction over time for spores collected by DEP, which also show noticeably higher average Bruker scores.

Two key measures within these datasets are the average Bruker spectral-comparison score over the entire time course (the higher this is, the more robustly the method will perform for identifications) and the average replicate-to-replicate spectra variation over the entire time course (the lower this is, the more reproducibly the method will perform). Replicate-to-replicate spectra variation can be measured as a coefficient of variation (CV, defined as the standard deviation divided by the mean, expressed as a percentage) between replicates. The effects of changing from biomass scraped from the agar to spores collected by DEP are shown in Table 1.

**Table 1:** The effects of changing from biomass scraped from the agar to spores collected by DEP

	Average cognate-medium Bruker score over the entire time course			Average cognate-medium CV over the entire time course		
*Penicillium* species	MEA	PDA	All media	MEA	PDA	All media
*Penicillium chrysogenum*	2.267–2.474	2.451–2.387	2.340–2.405	6.862–1.750	2.667–2.179	2.022–1.770
IMI 293188^a^^.^	improvement	reduction	improvement	improvement	improvement	improvement
*Penicillium corylophilum*	2.038–2.065	1.959–1.996	1.904–1.906	14.650–6.537	3.977–3.326	8.209–6.465
IMI 273248^b^^.^	improvement	improvement	improvement	improvement	improvement	improvement
*Penicillium digitatum*	2.119–2.175	2.366–2.312	2.131–2.186	4.407–5.786	2.894–2.183	6.169–3.972
IMI 380881	improvement	reduction	improvement	increase	improvement	improvement
*Penicillium glabrum*	2.005–1.970	2.314–2.402	2.078–2.079	6.939–3.506	4.973–1.857	5.432–3.403
IMI 320720	reduction	improvement	improvement	improvement	improvement	improvement
*Penicillium roqueforti*	1.990–2.629	2.372–2.673	2.142–2.576	5.987–0.882	2.666–0.655	3.603–1.212
IMI 297987^c^^.^	improvement	improvement	improvement	improvement	improvement	improvement

^a^ Discounting the 6-DAY MEA_CON test datapoints.

^b^ Discounting the 6-DAY MEA_CON, 6-DAY MEA_DEP, 10-DAY MEA_DEP, 17-DAY MEA_CON, 17-DAY MEA_DEP and 17-DAY PDA_CON test datapoints.

^c^ Discounting the 6-DAY MEA_CON test datapoints.

## Discussion and conclusions

Reasoning that, while protein expression in fungal mycelium may well vary under different growth conditions, fungal spores are essentially static dispersal and/or survival structures (which might be expected to vary less with growth conditions), we have developed a MALDI-TOF MS-based method for fungal identification through the analysis of acid-soluble proteins from spores collected using a rapid, inexpensive and simple dielectrophoretic method. This method for spore harvesting was selected because, for some fungi, the mycelium is anchored to the growth substrate and spores are physically-separated entities, and are, therefore, potentially amenable to a differential collection using DEP. Using non-uniform electrical fields conveniently generated through triboelectric charging of polystyrene microbiological inoculating loops, spores from five species from within the genus *Penicillium*, grown on agar plates containing two different media over an extended time course, have been investigated.

As much of our work is focused on reducing crop loss in Developing Countries, we chose to work initially with plant-associated fungi. While the spores collected in our study were strongly green pigmented, some fungal spores (e.g. human-pathogenic *Aspergilli*) are even more strongly pigmented – an additional challenge for MALDI-TOF MS analysis. Problems when working with such materials can, however, often be overcome by sample dilution, where the interference to the MALDI process ‘dilutes out’ faster than the ability to obtain a spectrum with good signal strength. As spectrometers have become more sensitive over time, even more dilution is often now possible before the spectra are too weak to detect.

The performance of the dielectrophoretic method has been assessed in 30 test cases. Overall, in terms of average Bruker spectral-comparison scores, changing from biomass scraped from the agar to spores collected by DEP led to higher scores in 12/15 (80%) of cases tested and lower scores in 3/15 (20%) of cases and, in terms of average CVs, changing from biomass scraped from the agar to spores collected by DEP led to lower variability in 14/15 (93%) of cases tested and greater variability in 1/15 (7%) of cases. Given that reducing variance is the key aim of this work (provided that identification scores do not fall sufficiently to impede identification), this is encouraging; in all but one case, the variance was reduced using the dielectrophoretic method, while higher Bruker spectral-comparison scores were also obtained 80% of the time. Finally, combining all 30 test results, changing from biomass scraped from the agar to spores collected by DEP led to the improvement in 26/30 (87%) of cases tested. For the five species investigated from within the genus *Penicillium*, grown on agar plates containing two different media over an extended time course, our method therefore facilitates improved identification with these species, which is an encouraging first step along the path to our ultimate goal of providing methods to facilitate the construction of spore-protein-based databases for fungal identification using MALDI-TOF MS. While the dielectrophoretic method offers some technical improvements, the spectral-similarity does nevertheless still decrease for all cases except *P. roqueforti* IMI 297987 on either medium with spores collected by DEP. This suggests that, with the exception of the latter, the spore-protein composition might change (albeit fairly minimally) over fungal-growth time; an indication of some residual biological variance in spite of our original rationale for selectively-analysing spores.

As an observation while carrying out the sample processing, the PDA medium appears to encourage sporulation as a powdery coating above the mycelium – a growth characteristic that makes spore harvesting very effective with the dielectrophoretic method. MEA is less effective in this regard, and spores appear to be more mixed in with the mycelium. This was particularly noticeable for *P. corylophilum* IMI 273248 and *P. glabrum* IMI 320720, with the former also showing a slight tendency towards spores mixed in with the mycelium even on PDA (e.g. see Supplementary Fig. S2, which shows the plates from the 6-day time point). While this was the case for the five Penicillium species studied, requirements for sporulation vary widely among fungi and low-nutrient media often produce better sporulation than sugar-rich media such as PDA because, when fungi are subject to difficult growing conditions, they often sporulate as a survival response.

A second observation is the extent to which spore-derived peaks appear to dominate the spectra of even the control method with biomass scraped from the agar. One possible explanation for this is that, after scraping the surface with an inoculating loop, mycelium is not harvested as efficiently as spores even when sporulation is light (e.g. at 3 days). Another possibility is that lysis and/or extraction prior to MALDI desorption and ionization is more efficient for spores that mycelium. Future work will aim to further investigate this observation.

In terms of limitations, this study was restricted to five representative fungal species from within the genus *Penicillium* on two media and so the utility of our method will now need testing with a wider variety of fungal genera and families, in conjunction with suitable culture media, in further investigations extrapolating from this successful initial proof-of-concept study. Key questions will be whether spore-protein spectra can distinguish beyond species level, whether our method is broadly applicable across different types of spores and what approach(es) will be needed in cases where more than a single type of spore might be produced during fungal culture. Further work will also endeavour to characterize better the method. Microscopic examination of harvested material could provide better insights into precisely what material is harvested, and in what proportions, and physical studies might possibly be able to delineate between the relative contributions from dielectrophoretic forces and contact forces after electrostatic attraction.

## Supplementary Material

bpz018_Supplementary_Data
