# Supplementary material for: Identification of Penicillium species by MALDI-TOF MS analysis of spores collected by dielectrophoresis
Source: Biol Methods Protoc. 2019 Nov 29;4(1):bpz018. doi: 10.1093/biomethods/bpz018 (PMC6994048; doi:10.1093/biomethods/bpz018)
Supplement: bpz018_Supplementary_Data [file bpz018_supplementary_data.pdf]

Supplementary materials

Table S1 Bruker spectral-comparison scores for *P. chrysogenum* IMI 293188 (CHR)

Reference spectrum	3 DAYS_MEA_CON_REP_2	3 DAYS_MEA_CON_REP_3	3 DAYS_MEA_DEP_REP_2	3 DAYS_MEA_DEP_REP_3	3 DAYS_PDA_CON_REP_2	3 DAYS_PDA_CON_REP_3	3 DAYS_PDA_DEP_REP_2	3 DAYS_PDA_DEP_REP_3	6 DAYS_MEA_CON_REP_2	6 DAYS_MEA_CON_REP_3	6 DAYS_MEA_DEP_REP_2	6 DAYS_MEA_DEP_REP_3	6 DAYS_PDA_CON_REP_2	6 DAYS_PDA_CON_REP_3	6 DAYS_PDA_DEP_REP_2	6 DAYS_PDA_DEP_REP_3
3 DAYS_MEA_CON_REP_1	2.530	2.525	2.114	2.199	2.450	2.392	2.109	2.071	2.310	-	2.092	2.073	2.429	2.351	2.144	1.950
3 DAYS_MEA_DEP_REP_1	2.578	2.455	2.607	2.717	2.524	2.598	2.571	2.544	2.226	-	2.561	2.528	2.210	2.366	2.227	2.278
3 DAYS_PDA_CON_REP_1	2.588	2.479	2.471	2.526	2.809	2.675	2.479	2.396	2.315	-	2.424	2.289	2.440	2.421	2.329	2.332
3 DAYS_PDA_DEP_REP_1	2.464	2.365	2.519	2.506	2.469	2.504	2.509	2.654	2.249	-	2.570	2.591	2.274	2.333	2.507	2.524

Reference spectrum	10 DAYS_MEA_CON_REP_2	10 DAYS_MEA_CON_REP_3	10 DAYS_MEA_DEP_REP_2	10 DAYS_MEA_DEP_REP_3	10 DAYS_PDA_CON_REP_2	10 DAYS_PDA_CON_REP_3	10 DAYS_PDA_DEP_REP_2	10 DAYS_PDA_DEP_REP_3	17 DAYS_MEA_CON_REP_2	17 DAYS_MEA_CON_REP_3	17 DAYS_MEA_DEP_REP_2	17 DAYS_MEA_DEP_REP_3	17 DAYS_PDA_CON_REP_2	17 DAYS_PDA_CON_REP_3	17 DAYS_PDA_DEP_REP_2	17 DAYS_PDA_DEP_REP_3
3 DAYS_MEA_CON_REP_1	2.098	2.506	2.343	2.449	2.440	2.433	2.443	2.412	2.038	1.822	2.100	1.990	2.094	2.133	1.931	1.956
3 DAYS_MEA_DEP_REP_1	2.429	2.418	2.371	2.401	2.447	2.471	2.480	2.400	2.426	2.168	2.266	2.340	2.269	2.318	2.161	2.174
3 DAYS_PDA_CON_REP_1	2.320	2.276	2.126	2.233	2.408	2.516	2.349	2.380	2.180	1.956	2.119	2.221	2.113	2.222	2.015	2.059
3 DAYS_PDA_DEP_REP_1	2.450	2.121	2.064	2.219	2.303	2.280	2.114	2.114	2.396	2.151	2.412	2.363	2.315	2.322	2.407	2.266

Table S2 Bruker spectral-comparison scores for *P. corylophilum* IMI 273248 (COR)

Reference spectrum	3 DAYS_MEA_CON_REP_2	3 DAYS_MEA_CON_REP_3	3 DAYS_MEA_DEP_REP_2	3 DAYS_MEA_DEP_REP_3	3 DAYS_PDA_CON_REP_2	3 DAYS_PDA_CON_REP_3	3 DAYS_PDA_DEP_REP_2	3 DAYS_PDA_DEP_REP_3	6 DAYS_MEA_CON_REP_2	6 DAYS_MEA_CON_REP_3	6 DAYS_MEA_DEP_REP_2	6 DAYS_MEA_DEP_REP_3	6 DAYS_PDA_CON_REP_2	6 DAYS_PDA_CON_REP_3	6 DAYS_PDA_DEP_REP_2	6 DAYS_PDA_DEP_REP_3
3 DAYS_MEA_CON_REP_1	2.442	2.409	2.137	2.154	2.334	2.359	2.169	2.274	2.114	-	2.269	-	2.172	2.234	1.811	1.690
3 DAYS_MEA_DEP_REP_1	2.390	2.049	2.218	2.433	2.327	2.375	2.332	2.133	2.153	-	2.103	-	2.029	2.190	1.662	1.769
3 DAYS_PDA_CON_REP_1	2.491	2.424	2.066	2.157	2.387	2.592	2.234	2.307	1.959	-	2.301	-	2.267	2.372	1.692	1.502
3 DAYS_PDA_DEP_REP_1	2.300	2.149	2.120	2.021	2.100	2.224	2.281	2.381	2.060	-	2.127	-	2.180	2.225	1.869	2.028

Reference spectrum	10 DAYS_MEA_CON_REP_2	10 DAYS_MEA_CON_REP_3	10 DAYS_MEA_DEP_REP_2	10 DAYS_MEA_DEP_REP_3	10 DAYS_PDA_CON_REP_2	10 DAYS_PDA_CON_REP_3	10 DAYS_PDA_DEP_REP_2	10 DAYS_PDA_DEP_REP_3	17 DAYS_MEA_CON_REP_2	17 DAYS_MEA_CON_REP_3	17 DAYS_MEA_DEP_REP_2	17 DAYS_MEA_DEP_REP_3	17 DAYS_PDA_CON_REP_2	17 DAYS_PDA_CON_REP_3	17 DAYS_PDA_DEP_REP_2	17 DAYS_PDA_DEP_REP_3
3 DAYS_MEA_CON_REP_1	1.259	1.890	-	-	1.882	1.816	1.736	1.756	-	-	-	1.586	-	1.000	1.654	1.633
3 DAYS_MEA_DEP_REP_1	1.296	1.729	-	-	1.928	1.809	1.904	1.590	-	-	-	1.767	-	0.959	1.737	1.404
3 DAYS_PDA_CON_REP_1	1.384	1.921	-	-	1.906	1.986	1.683	1.812	-	-	-	1.656	-	1.082	1.724	1.717
3 DAYS_PDA_DEP_REP_1	1.148	1.774	-	-	1.886	1.950	1.901	1.826	-	-	-	1.585	-	1.113	1.861	1.818

Table S3 Bruker spectral-comparison scores for *P. digitatum* IMI 380881 (DIG)

Reference spectrum	3 DAYS_MEA_CON_REP_2	3 DAYS_MEA_CON_REP_3	3 DAYS_MEA_DEP_REP_2	3 DAYS_MEA_DEP_REP_3	3 DAYS_PDA_CON_REP_2	3 DAYS_PDA_CON_REP_3	3 DAYS_PDA_DEP_REP_2	3 DAYS_PDA_DEP_REP_3	6 DAYS_MEA_CON_REP_2	6 DAYS_MEA_CON_REP_3	6 DAYS_MEA_DEP_REP_2	6 DAYS_MEA_DEP_REP_3	6 DAYS_PDA_CON_REP_2	6 DAYS_PDA_CON_REP_3	6 DAYS_PDA_DEP_REP_2	6 DAYS_PDA_DEP_REP_3
3 DAYS_MEA_CON_REP_1	2.480	2.347	2.286	2.330	2.281	2.351	1.828	1.803	2.392	2.419	2.094	1.973	2.192	2.232	1.773	1.733
3 DAYS_MEA_DEP_REP_1	2.337	2.128	2.450	2.273	2.427	2.402	2.289	2.370	2.350	2.363	2.342	2.211	2.224	2.405	2.157	2.016
3 DAYS_PDA_CON_REP_1	2.298	1.887	2.230	2.163	2.641	2.570	2.244	2.393	2.076	2.295	2.100	2.037	2.453	2.603	2.063	2.092
3 DAYS_PDA_DEP_REP_1	2.105	1.703	2.417	2.378	2.303	2.104	2.672	2.631	2.192	2.257	2.427	2.433	2.325	2.240	2.472	2.373

Reference spectrum	10 DAYS_MEA_CON_REP_2	10 DAYS_MEA_CON_REP_3	10 DAYS_MEA_DEP_REP_2	10 DAYS_MEA_DEP_REP_3	10 DAYS_PDA_CON_REP_2	10 DAYS_PDA_CON_REP_3	10 DAYS_PDA_DEP_REP_2	10 DAYS_PDA_DEP_REP_3	17 DAYS_MEA_CON_REP_2	17 DAYS_MEA_CON_REP_3	17 DAYS_MEA_DEP_REP_2	17 DAYS_MEA_DEP_REP_3	17 DAYS_PDA_CON_REP_2	17 DAYS_PDA_CON_REP_3	17 DAYS_PDA_DEP_REP_2	17 DAYS_PDA_DEP_REP_3
3 DAYS_MEA_CON_REP_1	2.158	2.189	1.832	1.782	1.961	2.030	1.805	1.713	1.607	1.357	1.687	1.649	1.724	1.736	1.604	1.715
3 DAYS_MEA_DEP_REP_1	2.260	2.357	1.979	2.187	2.135	2.290	1.639	1.886	1.797	1.007	1.886	2.074	2.084	2.103	1.697	1.932
3 DAYS_PDA_CON_REP_1	2.112	2.249	1.957	2.101	2.240	2.384	1.678	1.840	1.833	1.061	1.863	1.985	2.034	2.004	1.625	2.037
3 DAYS_PDA_DEP_REP_1	2.300	2.369	2.258	2.142	2.471	2.451	2.073	2.204	1.858	0.871	2.019	2.010	2.168	2.217	2.041	2.029

Table S4 Bruker spectral-comparison scores for *P. glabrum* IMI 320720 (GLA)

Reference spectrum	3 DAYS_MEA_CON_REP_2	3 DAYS_MEA_CON_REP_3	3 DAYS_MEA_DEP_REP_2	3 DAYS_MEA_DEP_REP_3	3 DAYS_PDA_CON_REP_2	3 DAYS_PDA_CON_REP_3	3 DAYS_PDA_DEP_REP_2	3 DAYS_PDA_DEP_REP_3	6 DAYS_MEA_CON_REP_2	6 DAYS_MEA_CON_REP_3	6 DAYS_MEA_DEP_REP_2	6 DAYS_MEA_DEP_REP_3	6 DAYS_PDA_CON_REP_2	6 DAYS_PDA_CON_REP_3	6 DAYS_PDA_DEP_REP_2	6 DAYS_PDA_DEP_REP_3
3 DAYS_MEA_CON_REP_1	2.527	2.489	1.929	2.099	2.312	2.301	1.833	1.681	1.911	2.092	1.813	2.091	2.180	1.750	1.677	1.720
3 DAYS_MEA_DEP_REP_1	2.223	2.056	2.125	2.216	2.140	2.165	1.987	1.929	2.253	2.334	2.067	2.001	2.121	1.905	1.941	1.951
3 DAYS_PDA_CON_REP_1	2.312	2.276	2.218	2.189	2.558	2.738	2.355	2.194	2.114	2.204	2.067	1.873	2.572	2.339	2.085	2.174
3 DAYS_PDA_DEP_REP_1	2.094	1.836	2.171	2.218	2.206	2.327	2.562	2.554	2.253	2.232	1.971	1.635	2.139	2.330	2.463	2.452

Reference spectrum	10 DAYS_MEA_CON_REP_2	10 DAYS_MEA_CON_REP_3	10 DAYS_MEA_DEP_REP_2	10 DAYS_MEA_DEP_REP_3	10 DAYS_PDA_CON_REP_2	10 DAYS_PDA_CON_REP_3	10 DAYS_PDA_DEP_REP_2	10 DAYS_PDA_DEP_REP_3	17 DAYS_MEA_CON_REP_2	17 DAYS_MEA_CON_REP_3	17 DAYS_MEA_DEP_REP_2	17 DAYS_MEA_DEP_REP_3	17 DAYS_PDA_CON_REP_2	17 DAYS_PDA_CON_REP_3	17 DAYS_PDA_DEP_REP_2	17 DAYS_PDA_DEP_REP_3
3 DAYS_MEA_CON_REP_1	1.887	2.118	1.557	1.619	1.631	1.741	1.428	1.621	1.639	1.380	1.489	1.433	1.339	1.510	1.685	1.372
3 DAYS_MEA_DEP_REP_1	2.101	2.127	1.855	1.729	1.815	1.744	1.657	1.667	2.024	1.967	1.933	1.832	1.504	1.632	1.673	1.405
3 DAYS_PDA_CON_REP_1	2.055	2.167	1.951	2.013	2.064	2.247	1.895	2.196	2.062	1.989	1.829	2.125	2.029	1.962	2.21	2.028
3 DAYS_PDA_DEP_REP_1	2.320	2.194	2.354	2.378	2.387	2.433	2.297	2.460	2.221	2.208	2.266	2.344	2.149	2.255	2.182	2.246

Table S5 Bruker spectral-comparison scores for *P. roqueforti* IMI 297987 (ROQ)

Reference spectrum	3 DAYS_MEA_CON_REP_2	3 DAYS_MEA_CON_REP_3	3 DAYS_MEA_DEP_REP_2	3 DAYS_MEA_DEP_REP_3	3 DAYS_PDA_CON_REP_2	3 DAYS_PDA_CON_REP_3	3 DAYS_PDA_DEP_REP_2	3 DAYS_PDA_DEP_REP_3	6 DAYS_MEA_CON_REP_2	6 DAYS_MEA_CON_REP_3	6 DAYS_MEA_DEP_REP_2	6 DAYS_MEA_DEP_REP_3	6 DAYS_PDA_CON_REP_2	6 DAYS_PDA_CON_REP_3	6 DAYS_PDA_DEP_REP_2	6 DAYS_PDA_DEP_REP_3
3 DAYS_MEA_CON_REP_1	2.307	2.098	2.078	2.011	2.056	2.183	1.799	1.761	2.017	-	1.841	1.884	2.26	1.974	1.773	1.633
3 DAYS_MEA_DEP_REP_1	2.508	2.697	2.705	2.631	2.482	2.453	2.536	2.522	2.586	-	2.587	2.601	2.382	2.477	2.512	2.468
3 DAYS_PDA_CON_REP_1	2.458	2.381	2.367	2.323	2.592	2.509	2.266	2.278	2.18	-	2.113	2.176	2.519	2.477	2.336	2.269
3 DAYS_PDA_DEP_REP_1	2.247	2.516	2.416	2.512	2.551	2.523	2.677	2.692	2.332	-	2.519	2.545	2.378	2.482	2.682	2.726

Reference spectrum	10 DAYS_MEA_CON_REP_2	10 DAYS_MEA_CON_REP_3	10 DAYS_MEA_DEP_REP_2	10 DAYS_MEA_DEP_REP_3	10 DAYS_PDA_CON_REP_2	10 DAYS_PDA_CON_REP_3	10 DAYS_PDA_DEP_REP_2	10 DAYS_PDA_DEP_REP_3	17 DAYS_MEA_CON_REP_2	17 DAYS_MEA_CON_REP_3	17 DAYS_MEA_DEP_REP_2	17 DAYS_MEA_DEP_REP_3	17 DAYS_PDA_CON_REP_2	17 DAYS_PDA_CON_REP_3	17 DAYS_PDA_DEP_REP_2	17 DAYS_PDA_DEP_REP_3
3 DAYS_MEA_CON_REP_1	1.971	1.791	1.946	1.956	1.808	1.826	1.804	1.814	1.920	1.802	1.992	1.907	1.689	1.779	1.896	1.764
3 DAYS_MEA_DEP_REP_1	2.650	2.517	2.639	2.661	2.494	2.511	2.526	2.438	2.556	2.408	2.615	2.593	2.394	2.403	2.495	2.366
3 DAYS_PDA_CON_REP_1	2.277	2.156	2.218	2.199	2.195	2.303	2.317	2.306	2.323	2.127	2.306	2.260	2.131	2.248	2.347	2.247
3 DAYS_PDA_DEP_REP_1	2.651	2.624	2.531	2.507	2.668	2.685	2.678	2.689	2.550	2.450	2.540	2.583	2.591	2.646	2.635	2.606

Figure S1

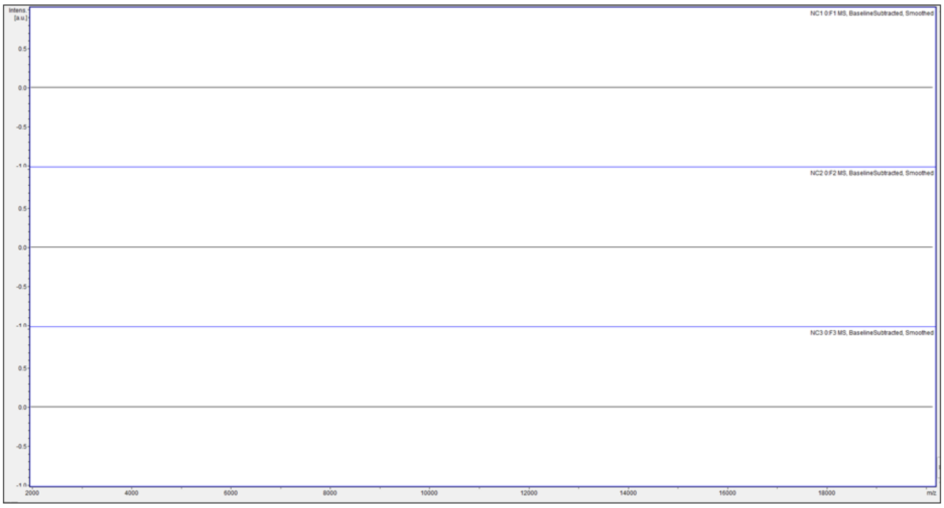

Figure S1 Negative-control MALDI-TOF MS spectra.

Figure S2

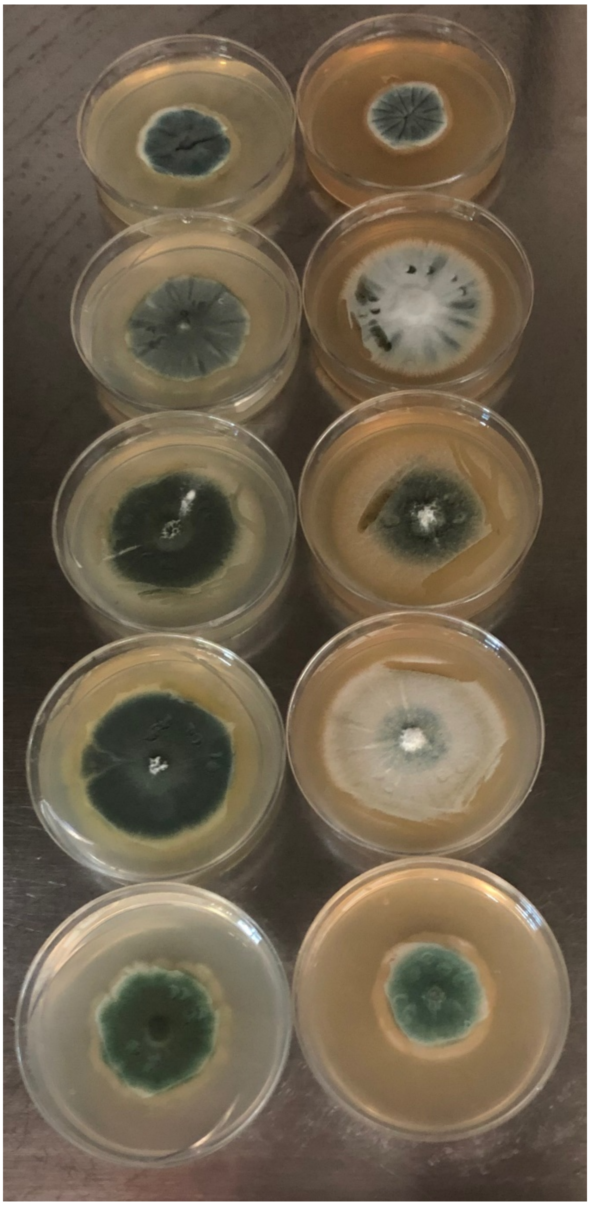

Figure S2 Agar plates from the 6-day time point showing, from top to bottom, *P. chrysogenum* IMI 293188, *P. corylophilum* IMI 273248, *P. digitatum* IMI 380881, *P. glabrum* IMI 320720, and *P. roqueforti* IMI 297987 and, on the left-hand side, growth on PDA and, on the right-hand side, growth on MEA.
